# Fluocinolone acetonide 0.19-mg implant for the treatment of noninfectious uveitis with involvement of the posterior segment: a real-world study

**DOI:** 10.1007/s00417-022-05893-2

**Published:** 2022-11-18

**Authors:** Lara Buhl, Stephan Thurau, Christoph Kern

**Affiliations:** Department of Ophthalmology, Ludwig-Maximilians-University, Mathildenstrasse 8, 80336 Munich, Germany

**Keywords:** Uveitis, Cystoid macular edema, Fluocinolone acetonide implant

## Abstract

**Purpose:**

To evaluate the effectiveness of 0.19-mg fluocinolone acetonide implant (FAi) for preventing inflammatory relapses in noninfectious uveitis with posterior segment involvement in standard clinical practice. Further, to assess the value of remission induction therapy with intraocular and periorbital administered high-dose corticosteroids before FAi.

**Methods:**

A retrospective cohort study in a tertiary referral center specialized in uveitis management. The primary study outcomes were the best-corrected visual acuity (BVCA) and central retinal thickness (CRT) within a 12-month observation period. The secondary outcomes were intraocular pressure (IOP) and intraocular inflammation. The main safety measures were IOP increase and cataract formation.

**Results:**

In total, 76 eyes of 57 patients received FAi. Locally administered high-dose corticosteroids were applied in 68.4% of all eyes before FAi. BCVA remained stable within the 12-month observation period (63.21 vs. 62.95, difference 0.26 letters; 95% CI: − 6.31 to 6.84; *p* > 0.9). Significant CRT reduction upon FAi was sustained after 12 months (362.7 vs. 309.1 μm, difference 53.57 μm; 95% CI: 1.55 to 105.6; *p* = 0.04). Intraocular inflammation was reduced until 9 months of follow-up (0.82 vs. 0.3, difference 0.53; 95% CI: 0.11 to 0.95; *p* = 0.007). A mean IOP increase (13.68 vs. 15.6; difference − 1.92; 95% CI: − 3.85 to 0.004; *p* = 0.0507) and cataract development (20% of all phakic eyes) were noted.

**Conclusion:**

We observed similar levels of FAi effectiveness for the treatment of noninfectious uveitis in standard clinical practice compared to previous randomized clinical trials. Moreover, remission induction therapy before FAi can benefit patients with increased baseline uveitis activity.

**Supplementary information:**

The online version contains supplementary material available at 10.1007/s00417-022-05893-2.

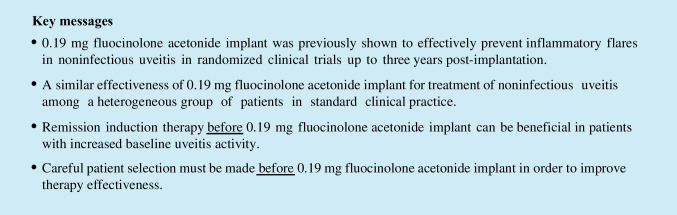

## Introduction

Noninfectious uveitis is a chronic disease characterized by recurrent inflammatory flares leading to accumulative retinal damage and irreversible vision loss in patients [1]. It accounts for up to 15% of cases of legal blindness in industrialized countries [2]. Thus, preventing inflammatory episodes is critical for maintaining patients’ long-term visual acuity and quality of life.

Corticosteroids administered topically, orally, or by intravitreal or periorbital injection are a mainstay in uveitis treatment [1, 2]. Corticosteroid implants, in particular, are well-established alternatives in patients who do not tolerate or respond to systemic immunomodulatory therapy and are especially helpful for treating persisting cystoid macular edema (CME). Up to now, four long-lasting corticosteroid implants have been available, either releasing dexamethasone or fluocinolone acetonide. The dexamethasone implant (Ozurdex, Allergan, Irvine, CA, USA) is a biodegradable implant that significantly improves visual acuity and reduces intraocular inflammation for three to six-month postinjection [3]. However, repeated treatment can be necessary with recurring disease flares and CMO [4]. In contrast, the first approved 0.59-mg fluocinolone acetonide implant (Retisert, Bausch & Lomb, Rochester, NY, USA) showed high effectiveness in diminishing recurrence rates of noninfectious posterior uveitis up to three years [5]. However, this implant caused increased ocular complications, such as elevated intraocular pressure (IOP), making pharmacotherapy and IOP-lowering surgery necessary, and enhanced cataract development [6, 7]. Most importantly, systemic immunosuppressive therapy was superior to the 0.59-mg fluocinolone acetonide implant regarding overall functional outcome after a seven-year follow-up [7].

More recently, a 0.19-mg fluocinolone acetonide implant (Iluvien, Alimera Sciences, Hampshire, UK; 0.18-mg fluocinolone acetonide implant by Yutiq EyePoint Pharmaceuticals, Inc., Watertown, MA, USA) was approved for the treatment of noninfectious uveitis affecting the posterior eye segment [1, 8]. Compared to the early 0.59-mg fluocinolone acetonide implant, the 0.19-mg fluocinolone acetonide implant (FAi) is intravitreally injected in an “in-office” procedure and emits a lower dosage to reduce corticosteroid-associated ocular complications [9]. It is approved to prevent inflammatory relapses in noninfectious uveitis with involvement of the posterior segment. Recent randomized clinical trials (RCTs) have demonstrated that FAi to significantly reduces uveitis recurrences up to three-year post-implantation [10–12]. However, the effectiveness of FAi for long-term uveitis control in a real-life setting has only been evaluated in small case series so far [13–15]. Therefore, in the present study, we sought to assess the efficacy of FAi for noninfectious uveitis in an extended real-world study at a tertiary referral center specialized in uveitis management.

This implant has been shown to prevent inflammatory relapses, implying that uveitis activity should be well controlled at the time of implantation. However, remission induction therapy before FAi was not further defined in recent trials [10–12]. In the present study, we applied two commonly used locally administered corticosteroids, an intravitreal 0.7 mg dexamethasone implant, and periorbital 40 mg triamcinolone acetonide three months and four weeks before FAi implantation for remission induction, respectively. Both therapies have previously been shown to effectively decrease uveitis activity [3, 16]. Therefore, we sought to investigate whether remission induction therapy before FAi is beneficial for long-term disease control and the prevention of recurrences.

## Methods

### Study design

This study was a retrospective cohort study conducted at the Department of Ophthalmology, Ludwig-Maximilians-University, Munich, Germany, evaluating the real-life efficiency of the 0.19-mg fluocinolone acetonide implant (FAi; Iluvien, Alimera Sciences, Hampshire, UK) in patients with noninfectious uveitis. The ethical approval was obtained from the Institutional Review Board of the Department of Ophthalmology at the Ludwig-Maximilians-University and the study protocol adhered to the Declaration of Helsinki.

**Fig. 1:**
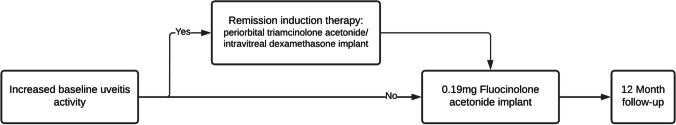
Treatment scheme

All patients were diagnosed with recurrent or chronic noninfectious uveitis with involvement of the posterior segment for at least three months. The reasons for FAi implantation were an insufficient response to systemic immunosuppressive therapy or intolerable side effects as well as a history of repeated relapses after local high-dose corticosteroids. Active inflammation was controlled either by the periorbital injection of 40 mg triamcinolone at four weeks or intravitreal 0.7 mg dexamethasone implantation (Ozurdex, Allergan, Irvine, CA, USA) at three months before the insertion of the FAi. The treatment approach was chosen according to the severity of the initial inflammation. Patients with increased intraocular inflammation received a dexamethasone implant for a more potent and sustained immunosuppressive effect. Data were obtained at baseline and at 3, 6, 9, and 12 months after FAi implantation. The broad treatment scheme is depicted in Fig. 1.

### Study outcomes

The primary study outcomes were the best-corrected visual acuity (BVCA) and central retinal thickness (CRT) as assessed by spectral-domain optical coherence tomography (OCT) (Spectralis OCT, Heidelberg Engineering, Heidelberg, Germany). The BCVA was defined as the best visual acuity value available for the visit and was exported as an Early Treatment Diabetic Retinopathy Study (ETDRS) letter score. If only decimal values were available, it was transformed into an EDTRS letter score, following an established method [17]. Secondary outcomes were intraocular pressure measurement (Goldmann or air-puff tonometry), assessment of intraocular inflammation by slit-lamp examination, FAi-associated complications, and disease recurrence during the 12-month observation period. Intraocular inflammation was assessed using the Standardization of Uveitis Nomenclature (SUN) classification [18]. Complications were defined as increased intraocular pressure (IOP), a need for IOP-lowering topical treatment or surgery, hypotony following injection, cataract formation, and any incident requiring surgical removal of the implant. Overall disease activity was assessed in consideration of all clinical findings, including BCVA, CRT, and SUN grading. Relapse was defined as the need for retreatment with intravitreal/periorbital corticosteroids.

### Statistical analysis

The data were collected with Microsoft Excel (Version 16.23 for Mac; Microsoft, Redmond, WA, USA). Statistical analysis was performed using GraphPad Prism 8 (La Jolla, CA; USA). The results are presented as mean values, including standard deviation (mean ± SD). The level of statistical significance was set at 0.05. Primary and secondary endpoint data were analyzed by intention to treat. To investigate the statistical significance of intra- and intergroup differences, a mixed-effects model was applied. Pearson’s correlation coefficient was calculated to compare CRT change (ΔCRT) with the respective BCVA change (ΔBCVA).

## Results

### Patient characteristics

Our cohort included 76 eyes and 58 patients who underwent 0.19-mg fluocinolone acetonide (FAi) implantation. In total, 44 patients (52 eyes) completed the 12-month observation period. Detailed baseline patient characteristics are depicted in Table 1. In total, 67 eyes (88.2%) had a history of periorbital or intravitreal corticosteroid injections. In our cohort 52 eyes (68.4%) received steroids for remission induction before FAi: either an intravitreal 0.7 mg dexamethasone implant (*n* = 35; 46.1%) or a periorbital injection of 40 mg triamcinolone acetonide (*n* = 17; 22.4%). We observed a significant reduction in CRT, intraocular inflammation (SUN grade), and a consecutive increase in BCVA upon remission induction therapy (Supplemental Fig. 1). In total, 24 eyes (31.6%) did not receive any induction therapy. Table 2 summarizes FAi patients’ characteristics with and without remission induction therapy within the 12-month observation period.

**Table 1:** Baseline patient characteristics

Number of eyes	76
Number of patients	58
Mean age ± SD (years)	53.5 ± 17.6
Sex (*n*; %)	
Female	62; (81.6)
Male	14; (18.4)
Uveitis segment involvement (*n*; %)	
Anterior	23; (30.3)
Intermedia	24; (31.6)
Posterior	24; (31.6)
Panuveitis	5; (6.6)
Mean BCVA ± SD (EDTRS letters)	63.2 ± 17.5
Mean IOP ± SD (mmHg)	13.7 ± 4.1
Lens status (n; %)	
Phakic	20; (26.3)
Pseudophakic	50; (65.8)
Aphakic	6; (7.9)
Systemic immunosuppressive therapy (*n*; %)	36; (47.4)

**Table 2:** Comparison of patients with and without remission induction therapy before 0.19-mg fluocinolone acetonide implantation

	Without remission induction therapy	With remission induction therapy
Number of eyes	24	52
Number of patients	19	39
Mean age (years) ± SD	51.7 ± 18.8	55.1 ± 16.9
Mean BCVA ± SD (EDTRS letters)		
At baseline	60.4 ± 17.3	64.5 ± 17.1
At month 12	64.7 ± 17.9	64.5 ± 17.6
Mean IOP ± SD (mmHg)		
At baseline	13.6 ± 3.2	13.7 ± 4.5
At month 12	15.3 ± 5.5	13.9 ± 5.5
IOP events within 12 months		
> 25 mmHg (*n*)	1	2
IOP-lowering medication at month 12 (*n*; %)	9; (37.5)	22; (42.3)
IOP-lowering surgery (*n*)	0	2
Recurrences within 12 months (*n*)	0	7

### Best-corrected visual acuity outcome

**Fig. 2:**
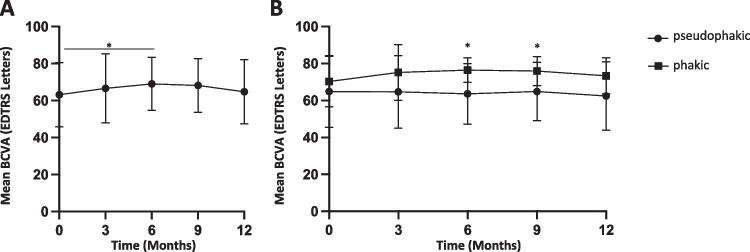
Mean change in best-corrected visual acuity (BCVA) after FAi implantation. (**A**) Mean BCVA change of all eyes included. (**B**) Comparison of mean BCVA change of pseudophakic and phakic eyes. *p < 0.05

Overall, the BCVA remained stable upon FAi implantation during the 12-month observation period (63.21 vs. 62.95 letters, mean difference 0.26 letters, 95% CI: − 6.31 to 6.84, *p* > 0.9) (Fig. 2A). The initial BCVA improvement within the first six months (63.21 vs. 68.78 letters, mean difference − 5.57 letters, 95% CI: 10.3 to − 0.84, *p* = 0.014) was not retained. The baseline BCVA differed in phakic and pseudophakic eyes (70.3 vs. 64.89 letters, difference − 5.41, 95% CI: − 17.21 to 6.39, *p* = 0.72). However, no significant BCVA change was observed within both groups over time (12 months compared to baseline in phakic eyes, 70.3 vs. 75.29 letters, difference − 4.99, 95% CI: − 14.71 to 4.72, *p* = 0.79; pseudophakic eyes, 64.14 vs. 63.18; difference 0.96, 95% CI: − 4.94 to 6.86, *p* > 0.9) (Fig. 2B). Both remission induction therapy and additional systemic therapy did not significantly affect BCVA outcome after 12 months (64.73 vs. 64.49 letters, difference 0.24, 95% CI: − 15.66 to 16.13, *p* > 0.9; 68.59 vs. 63.83, difference 4.77, 95% CI: − 6.69 to 16.23, *p* = 0.81) (Fig. 4A; Table 2).

### Macular edema

**Fig. 3:**
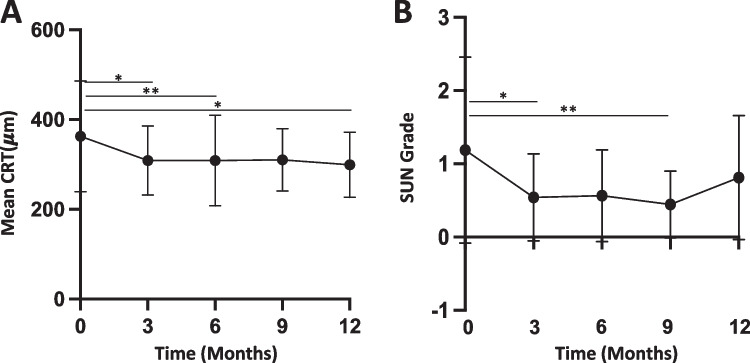
Control of inflammation after FAi implantation. (**A**) Mean change in central retinal thickness (CRT). (**B**) Overall intraocular inflammation according to SUN grading. *p < 0.05, **p < 0.01

**Fig. 4:**
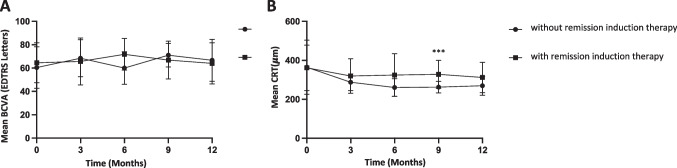
Comparison of patients with and without remission induction therapy before FAiimplantation. (**A**) Mean change of BCVA. (**B**) Mean change of CRT. ***p < 0.001

The mean central retinal thickness (CRT) significantly improved after FAi at three months compared to baseline (362.7 vs. 308.7 μm, difference 54.01 μm, 95% CI: 8.86 to 99.16, *p* = 0.01) and remained stable throughout the observation period (12 months compared to baseline, 362.7 vs. 309.1, difference 53.57 μm, 95% CI: 1.55 to 105.6, *p* = 0.04) (Fig. 3A). The change in CRT (ΔCT = baseline CRT – CRT after 3 months) did not significantly correlate with BCVA change (ΔBCVA = baseline BCVA – BCVA after 3 months; *r* =  − 0.099, *p* = 0.5) (Supplemental Fig. 2). Baseline CRT did not differ in FAi patients with or without remission induction therapy (364.5 vs 361.9 μm, difference 2.62 μm, 95% CI: − 85.79 to 91.02, *p* > 0.9). After 9 months, CRT was significantly reduced in patients without remission induction therapy compared to those with remission induction therapy (262.5 vs. 341.9 μm, difference − 79.4 μm, 95% CI: − 130 to − 28.75, *p* = 0.0007); however, this effect was not retained up to month 12 (278.8 vs. 322.9 μm, difference − 44.14 μm, 95% CI: − 99.36 to 11.08, *p* = 0.17) (Fig. 4B). No significant CRT difference in patients with additional systemic anti-inflammatory therapy was observed after 12 months (307.9 vs. 293.3 μm, difference 14.59 μm, 95% CI: − 55.51 to 84.68, *p* = 0.98).

### Inflammation

The overall intraocular inflammation assessed by SUN score initially improved after FAi at three months (0.83 vs. 0.18, difference 0.65, 95% CI: 0.26 to 1.04, *p* = 0.0001) (Fig. 3B) and remained stable until nine months of follow-up (0.83 vs. 0.3, difference 0.53, 95% CI: 0.11 to 0.95, *p* = 0.007). However, a slight, but not significant increase was observed after 12 months (0.83 vs. 0.55, difference 0.28, 95% CI: − 0.23 to 0.79, *p* = 0.53). Neither remission induction nor additional systemic therapy significantly affected intraocular inflammation (0.47 vs. 0.59, difference − 0.12, 95% CI: − 0.78 to 0.55, *p* = 0.9; 0.54 vs. 0.57, difference − 0.03, 95% CI: − 0.62 to 0.55, *p* > 0.9). It is noteworthy that intraocular inflammation was not the justifying indication for FAi in our cohort.

### Adjunctive immunosuppressive treatment

Overall, 36 eyes (47.4%) received adjunctive immunosuppressive treatment at baseline. In seven eyes (19.4% of all eyes that received adjunctive treatment and five patients in total), systemic adjunctive therapy was either stopped or reduced within the 12-month observation period. In three eyes, systemic adjunctive treatment was escalated. Some patients suffered from a systemic autoimmune disease requiring immunosuppressive therapy. Therefore, treatment was adjusted according not only to the ocular disease, but also to systemic activity.

### Recurrence rate

Within the 12-month observation period, seven recurrences (9.2%) with the need for subsequent treatment either with a 0.7 mg dexamethasone intravitreal implant (*n* = 6) or periorbital 40 mg triamcinolone acetonide were recorded (*n* = 1). Two eyes relapsed within three months after the implantation of FAi, suggesting that the initial disease activity exceeded the implant’s anti-inflammatory effect. Notably, recurrences were only reported in eyes that had undergone previous remission induction therapy (Table 2).

### Intraocular pressure and cataract formation

IOP did not significantly increase upon FAi treatment after 12 months; however, a tendency was observed (13.68 vs. 15.6 mmHg, difference − 1.92, 95% CI: − 3.85 to 0.004, *p* = 0.0507). An IOP rise of > 25 mmHg occurred in three eyes. Filtering procedures were performed in two eyes within the 12-month observation period (2.6%). IOP-lowering medication was administered in 26.3% of all eyes at the time of FAi implantation, and this increased to 31.6% after 12 months. The mean number of IOP-lowering medications slightly increased from 0.5 to 0.6 at baseline and after 12 months, respectively. Cataract surgery was required in 4 of the 20 phakic eyes. One eye underwent cataract surgery shortly after FAi implantation.

### Implantation-associated adverse events

Major implantation-associated adverse events included hypotony in seven cases (9.2%), which was reversible in all affected eyes, one vitreous hemorrhage (1.3%), and two anterior chamber dislocations (2.6%). Both affected eyes had a history of vitreoretinal surgery. One patient previously underwent replacement of the intraocular lens (IOL) with an iris-fixated IOL. In one case, FAi was explanted after recurring anterior chamber dislocation.

## Discussion

The study objective was to assess the safety and efficacy of 0.19-mg fluocinolone acetonide implant (FAi) for the treatment of noninfectious posterior uveitis in standard clinical practice. Moreover, we evaluated the potential benefits of high-dose corticosteroids for remission induction before FAi.

We observed statistically significant CRT reduction and functional stabilization after FAi implantation within the 12-month observation period. Seven recurrences requiring re-treatment were noted. Cataract surgery was necessary for 20% of all the phakic eyes. We noticed an increase in mean IOP with the necessity for the increased application of IOP-lowering medication and the need for filtering surgery in two eyes. A similar functional and morphological outcome was observed in FAi patients with and without remission induction therapy.

With randomized controlled trials being the gold standard when testing the efficacy of therapies, real-world studies still represent a valuable tool used to evaluate their effectiveness in everyday clinical practice [19]. Overall, we observed similar levels of FAi effectiveness in treating uveitis with involvement of the posterior segment compared to prior RCTs [10, 12]. Favorable effects on visual acuity and disease activity, including ocular inflammation and macular edema, were noted. Interestingly, we observed a recurrence rate lower than previously described [12]. This supposed difference, however, is biased by different recurrence criteria [12]. Nevertheless, remission induction therapy with either dexamethasone intravitreal implant or periorbital triamcinolone acetonide might also affect the remission rate within 12 months according to baseline uveitis activity.

The treatment regimen for noninfectious uveitis is commonly adjusted according to disease activity and inflammatory flares. In our cohort, patients received the FAi when remission could not be sustained by systemic therapy or short-acting local corticosteroids only. However, FAi was usually not considered as an alternative but an addition to systemic therapy. Consequently, the outcome observed can be confounded by adjunctive systemic corticosteroid and immunosuppressant therapy. Nevertheless, we did not note a significant difference in functional and morphological outcomes in FAi patients receiving conjunctive systemic anti-inflammatory treatment. One potential reason could be that, in most patients, FAi was administered to prevent macular edema recurrence, which was nonresponsive to systemic therapy despite sufficient inflammation control. Still, long-term results will be required to evaluate the potential benefit of systemic treatment additional to FAi.

Treating inflammatory flares with locally administered short-acting corticosteroids such as dexamethasone intravitreal implant or periorbital triamcinolone acetonide is common practice in uveitis management [3, 16]. These corticosteroids have already been used to silence disease activity before FAi [20]. However, the authors have no knowledge of randomized clinical trials comparing different remission induction modalities before FAi. Here, we show similar BCVA and CRT outcomes 12 months after FAi, comparing eyes treated with and without remission induction therapy with comparable side effects. The necessity for remission induction therapy, however, implies an increased uveitis activity at baseline in these eyes. Therefore, our data suggest that patients with increased uveitis activity can be treated with FAi with similar effectiveness as when treated with high-doses of short acting locally administered corticosteroids beforehand. Notably, we observed a higher recurrence rate in this subgroup than in those with well-controlled disease activity. Therefore, to optimize therapy effectiveness, careful patient selection must be made before FAi.

Macular edema is considered a leading cause of vision loss in uveitis [21]. Thus, increased retinal thickness was previously associated with poor visual acuity [22]. In the present study, we observed stabilization and further visual acuity improvement upon FAi implantation. However, the correlation of the BCVA increase with CRT reduction after 3 months was insignificant, suggesting that further improvement was not primarily the result of macula edema reduction but attenuation of intraocular inflammation and subsequent photoreceptor restoration. One reason may be that macula edema was mainly resolved at the time of FAi implantation due to remission induction therapy.

Secondary glaucoma is a common complication of uveitis [23]. Therefore, a considerable number of patients included in our cohort were treated with IOP-lowering medication (*n* = 24; 31.5%) or had a history of filtering surgery (*n* = 3; 3.9%) at baseline. As expected, the need for IOP-lowering medications increased within the observation period. Two eyes underwent filtering surgery. Overall, our results resemble previous findings after a 12-month observation period [10]. Similarly, cataract formation with subsequent cataract surgery was observed in the phakic group. However, BCVA in phakic eyes remained stable in our cohort. It is noteworthy that, in phakic patients, consecutive cataract surgery after intravitreal corticosteroid injection is common practice, naturally impeding the interpretation of the overall visual acuity outcome. In our cohort, one patient underwent cataract surgery shortly after FAi implantation.

Noninfectious uveitis is the collective term for various intraocular inflammatory diseases resulting from an aberrant autoimmune response to uveal structures associated with tissue destruction and a decline in visual acuity [24]. This study reflects the heterogeneity of patients receiving FAi in routine clinical practice. Patients with noninfectious uveitis of all segments and etiologies were analyzed, including isolated autoimmune entities, such as Fuchs’ iridocyclitis, multifocal choroiditis, birdshot chorioretinopathy, and entities associated with systemic diseases, such as Behçet’s disease, Vogt Koyanagi Harada’s disease, ankylosing spondylitis, collagen vascular diseases, and juvenile rheumatoid arthritis. However, not only did uveitis segment type or etiology differ, but history, comorbidities, and visual acuity at baseline varied widely in our cohort. Although an analysis of the different uveitis types or severities was not possible due to the limited number of patients, the overall effectiveness of FAi was observed. Nevertheless, this complexity surely complicates the subsequent analysis of treatment effectiveness. For instance, Ajamil-Rodanes et al. suggested choroidal inflammation not be fully responsive to low-dose intravitreal corticosteroid therapy due to insufficient penetration, highlighting the need for further subgroup analysis to identify clinical measures that favor responses to treatment [20].

This is a retrospective study conducted during routine clinical practice. Therefore, the digital patient data collected were occasionally inconclusive or incomplete, possibly altering our results. Additionally, an increased loss of follow-up was observed. Low follow-up adherence can be associated with inadequate treatment response and the subsequent transfer of care or absence of symptoms and remission. From our clinical experience, follow-ups are usually carried out by the primary care doctor, and patients are referred in cases of the disease worsening. With this, the recurrence rate can be overestimated in our cohort. As reported, many kinds of noninfectious uveitis were included in this study. However, the absolute number of eyes for each entity was deficient.

In conclusion, we observed the effectiveness of FAi for the treatment of noninfectious uveitis of the posterior segment, similarly to previous RCTs. Moreover, we found evidence that prior remission induction therapy can benefit patients with increased baseline uveitis activity. Indeed, a further follow-up is needed to evaluate the long-term efficacy of FAi for preventing inflammatory flares and visual acuity in our cohort. In future studies, subgroup analysis will inevitably define patient eligibility criteria for treatment.

## Supplementary information

Below is the link to the electronic supplementary material.

Supplementary file1 (PDF 50 KB)

Supplementary file2 (PDF 64 KB)
