# Supplementary figures and images for: Fluocinolone acetonide 0.19-mg implant for the treatment of noninfectious uveitis with involvement of the posterior segment: a real-world study
Source: Graefes Arch Clin Exp Ophthalmol. 2022 Nov 18;261(4):1101–8. doi: 10.1007/s00417-022-05893-2 (PMC10050064; doi:10.1007/s00417-022-05893-2)

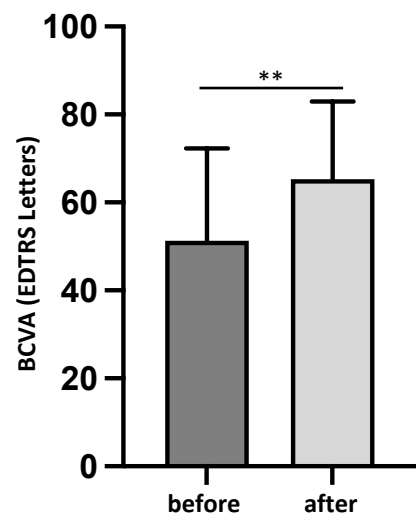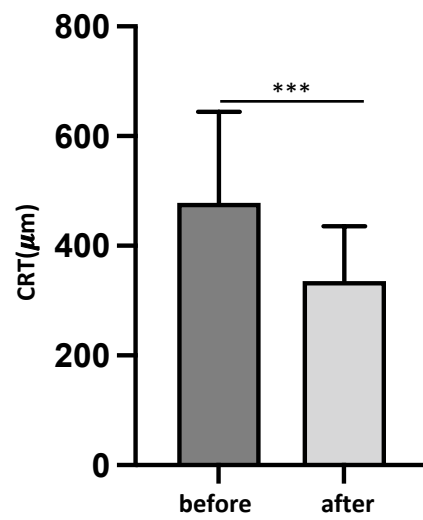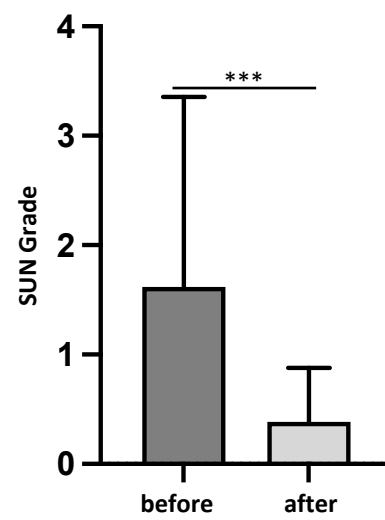

Supplement: Supplementary file 1 — Supplementary file1 (PDF 50 KB) [file 417_2022_5893_MOESM1_ESM.pdf]

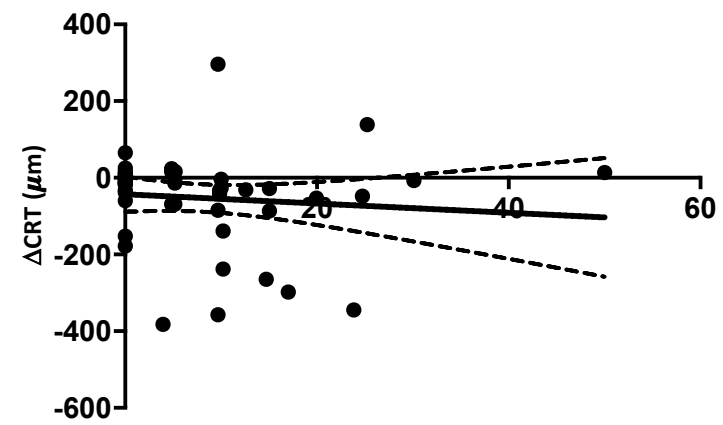

Supplement: Supplementary file 2 — Supplementary file2 (PDF 64 KB) [file 417_2022_5893_MOESM2_ESM.pdf]
